# Expiratory central airway collapse in stable COPD and during exacerbations

**DOI:** 10.1186/s12931-017-0646-2

**Published:** 2017-08-25

**Authors:** Paul Leong, Anne Tran, Jhanavi Rangaswamy, Laurence E. Ruane, Michael W. Fernando, Martin I. MacDonald, Kenneth K. Lau, Philip G. Bardin

**Affiliations:** 1 Monash Lung and Sleep, Monash Medical Centre, 246 Clayton Road, Clayton, 3168 Australia; 2 Monash University, Clayton, VIC Australia; 3 Diagnostic Imaging, Monash Medical Centre, Clayton, Australia

**Keywords:** COPD, Trachea, Ct

## Abstract

**Background:**

Tracheal obstruction resulting from expiratory tracheal deformation has been associated with respiratory symptoms and severe airway exacerbations. In chronic obstructive pulmonary disease (COPD), acute exacerbations (AECOPD) create large intrathoracic pressure swings which may increase tracheal deformation. Excessive central airway collapse (ECAC) may be diagnosed when the tracheal area on expiration is less than 50% of that on inspiration. The prevalence of ECAC in AECOPD and its temporal course have not been systematically studied.

**Methods:**

We prospectively recruited healthy volunteers (*n* = 53), stable outpatients with COPD (*n* = 40) and patients with hospitalised acute exacerbations of COPD (AECOPD, *n* = 64). 17 of the AECOPD group returned for repeat evaluation when clinically well at 6–12 weeks. All subjects underwent dynamic 320-slice computed tomography of the larynx and trachea during tidal breathing, enabling quantitation of tracheal area and dimensions (mean ± SD).

**Results:**

No healthy individuals had ECAC. The prevalence of ECAC in stable COPD and AECOPD was 35% and 39% respectively. Mean tracheal collapse did not differ between stable COPD (57.5 ± 19.8%), AECOPD (53.8 ± 19.3%) and in the subset who returned when convalescent (54.9 ± 17.2%). AECOPD patients with and without ECAC had similar clinical characteristics.

**Conclusions:**

Tracheal collapse in both stable and AECOPD is considerably more prevalent than in healthy individuals. ECAC warrants assessment as part of comprehensive COPD evaluation and management. Further studies should evaluate the aetiology of ECAC and whether it predisposes to exacerbations.

**Electronic supplementary material:**

The online version of this article (10.1186/s12931-017-0646-2) contains supplementary material, which is available to authorized users.

## Background

Fixed tracheal obstruction is an important cause of airflow obstruction, dyspnoea, cough, and sputum retention. During expiration, however, dynamic tracheal obstruction resulting from expiratory central airway collapse (ECAC) may result in similar degrees of obstruction [1–5]. This phenomenon has attracted limited attention, chiefly because of a lack of accurate tools to investigate and quantify real-time tracheal narrowing.

The nomenclature used to denote dynamic tracheal obstruction has been complex. Tracheobronchomalacia (TBM) describes narrowing from weakening of the tracheal cartilage, with a reported prevalence of 9% in an obstructive airways disease cohort [6]. Tracheal collapse from exaggerated posterior membrane invagination is termed excessive dynamic airway collapse (EDAC), and a greater than 50% reduction in tracheal area has been considered diagnostic [7–9]. Expiratory central airway collapse (ECAC) is an umbrella term referring to TBM or EDAC [10].

ECAC can occur frequently, impact pulmonary function and may be of considerable importance in diseases such as chronic obstructive pulmonary disease (COPD). For example, in the COPDGene cohort ECAC was associated with more respiratory symptoms, worse dyspnoea and more frequent exacerbations in smokers. ECAC may be of particular relevance in acute exacerbations of COPD (AECOPD) since exacerbations are accompanied by acute pathophysiological derangements and intrathoracic pressure changes that favour tracheal collapse [11]. To what extent ECAC is induced during AECOPD is currently not known and there have been no prospective investigation of the prevalence or clinical associations of ECAC in AECOPD. Presentations as varied as cough, dyspnoea, recurrent infections and difficulty weaning from respiratory support have been ascribed to the presence of EDAC [8] and accurate diagnosis may therefore have aetiological, prognostic and management implications in COPD [8].

The current diagnostic gold standard for ECAC is bronchoscopy, but precise quantification of collapse is difficult, confounded by optical distortion, sedation and airway instrumentation [12]. Recent developments in dynamic computerised tomography (CT) technologies provide a non-invasive, objective method to quantify tracheal movement and abnormalities [5, 13]. Several studies have employed dynamic CT to detect ECAC, most commonly during a forced expiration manoeuvre [2–4].

For the current study we hypothesized ECAC would be detected more often in COPD than in healthy individuals and that ECAC would be worsened during AECOPD. An exploratory, prospective, observational study was conducted during tidal breathing, using dynamic CT image analyses.

## Methods

### Patients

Upper airway CT was done in healthy volunteers (*n* = 53) without respiratory symptoms, any prior diagnosis of chest disease and not taking any respiratory medications. Stable patients with COPD (*n* = 40) were recruited from outpatient clinics. COPD was defined per GOLD criteria (postbronchodilator FEV_1_/FVC <0.70) with smoking history >10 pack years [14]. Stability was defined as the absence of exacerbations of COPD necessitating hospital admission, oral corticosteroids or antibiotics during the previous 3 months. Ethics approval was obtained from Monash Health Human Research Ethics Committee A (approval 12120A) and the study was conducted in accordance with the amended Declaration of Helsinki. All subjects provided verbal and written consent. This study did not meet the ICMJE/WHO clinical trial definition so registration was not required.

Patients requiring hospital admission to Monash Medical Centre, Melbourne, Australia for doctor-diagnosed AECOPD (*n* = 64) had upper airway CT conducted within 48 h of hospital admission following informed consent. Inclusion criteria for this group were age 40–90 years, a known diagnosis of COPD or a history of >10 pack years smoking with likely COPD (subsequently spirometrically confirmed). All patients had a history of deterioration in COPD symptoms leading to ED admission and severity was classified according to BAP-65 [15]. Exclusion criteria were known tracheal or laryngeal disease, a history of asthma, the inability to be recumbent for 10 min and known obstructive sleep apnoea: these exclusions also applied to the stable COPD group. A subgroup (*n* = 17/64, 26%) was reassessed 6–8 weeks after AECOPD and upper airway CT repeated.

Spirometry was performed according to ATS/ERS standards [16]. For the AECOPD group spirometry was not attempted during the acute event and values are as reported in the year preceding hospitalisation or when convalescent.

### Dynamic CT and analyses

Imaging was undertaken using 320-slice dynamic multidetector computerised tomography (Toshiba Aquilion ONE, Vision edition, Toshiba medical systems, parameters: 80kVp, 300 to 350 mA, 0.5 mm collimation, 0.275 s gantry rotation) as previously detailed [5]. Briefly, images were acquired in the supine position during tidal breathing, capturing approximately 2.5 respiratory cycles with a radiation dose of 0.5–1.5 mSv. Volume data over 16 cm from the superior larynx to the proximal mainstem bronchi was acquired. Integrated software programs were used to acquire continuous dynamic axial, sagittal, and coronal multiplanar images as well as dynamic 3D airway views using volume-rendering techniques. Images were reconstructed and could be viewed in cine mode. It was therefore possible to review the entire length of the trachea during both inspiration and expiration to identify ECAC (as well as distinguish TBM and EDAC) and to quantify its severity.

During ECAC, maximal airflow obstruction occurs at the narrowest part of the trachea (at the flow limiting segment or ‘choke point’ [1]). To ascertain this location, 4-dimensional volume rendering images were first reviewed in real-time and the narrowest point or region identified by visual inspection. To minimise partial voluming, images were formatted so the trachea was perpendicular to axial images and detailed analysis was undertaken at the narrowest level. Using integrated electronic tools (Phillips IntelliSpace v6.0.2.32500, 13 June 2014), luminal areas, anterior-posterior and lateral dimensions were measured on every patient at the narrowest point. ECAC was quantified by the ratio of minimum luminal tracheal area (expiration) to maximum tracheal area (inspiration). Anterior-posterior and lateral airway dimensions were similarly calculated.

To distinguish TBM and EDAC four-dimensional volume rendering sequences and axial views were compared to Murgu and Colt’s tracheal morphology classification [8, 17]. Tracheal shape per this schema was assessed as normal, TBM (sabre, concentric, crescentic) or EDAC. All cases were reviewed by two investigators (KL, PL) blinded to clinical data.

### Statistical analysis

Statistical analysis was conducted with IBM SPSS 20 (Armonk, NY, USA) with results shown as mean ± standard deviation (m ± SD). Pre-determined tests were used and were two-tailed where applicable: t-tests for unpaired parametric data, Mann-Whitney U for unpaired nonparametric data, paired t-test for paired parametric data, Wilcoxon signed-rank test for unpaired nonparametric data and Fisher’s exact test for categorical variables. Normality was judged by inspection of data plots and Kolmogorov–Smirnov tests. Significance was pre-specified at *p* < 0.05.

## Results

Subject characteristics are detailed in Tables 1 and 2. Subjects who completed enrolment including CTs were as follows: 53 healthy individuals, 40 patients with stable COPD and 64 patients with AECOPD. At 6–12 weeks after AECOPD repeat upper airway CT was done in 17 patients. Patients with COPD were slightly older than healthy participants. 8 subjects were consented for the AECOPD group but did not proceed to CT scan: CT maintenance (*n* = 4), unexpected breathlessness when lying down before imaging (*n* = 2), intractable cough (*n* = 1), withdrawal of consent (*n* = 1). All CTs were completed within 24 h of hospital admission (mean admission to scan time 12 h 29 min ± 7 h 38 min).

**Table 1:** Demographics of healthy individuals and stable COPD

	Healthy Individuals (*n* = 53)	Stable COPD (*n* = 40)	*P* value
Age, years	56.6 ± 16.9	70.1 ± 8.2	<0.001
Sex, (female:male)	18:35	21:19	
Body mass index (kg/m^2^)	30.2 ± 7.0	28.7 ± 6.3	0.273
Pack years smoked	9 ± 15	48.6 ± 39.5	<0.001
FEV_1_ (% predicted)	109 ± 17	65.7 ± 27.8	<0.001
FEV_1_/FVC	80 ± 10	55.4 ± 18.2	<0.001
GOLD airflow limitation class (*n*)	N/A	I 11 II 14 III 11 IV 4	n/a
Bronchodilator response (% change)	3 ± 4	8.9 ± 7.9	<0.001
TLCO (% predicted)	78 ± 15	51 ± 31.3	<0.001
Medications (*n*)			
• Prednisolone 1-10 mg/d	0	0	n/a
• Prednisolone >10 mg d	0	2	0.099
• Inhaled CS < = budesonide 800 mg/d	0	6	0.003
• Inhaled CS > 800 mg/d	0	18	<0.001
• LABA	0	23	<0.001
• LAMA	0	22	<0.001
Exacerbations requiring hospital admission in preceding year (*n*)	0	0.1 ± 0.38	<0.001

*FEV*_*1*_ forced expiratory volume in one second; *FVC* forced vital capacity; *TLCO* transfer capacity for carbon monoxide; *Inhaled CS* inhaled corticosteroid; *LABA* long active beta agonist; *LAMA* long acting muscarinic antagonist. Data are mean ± SD unless otherwise specified. *P* values are for independent samples t-test, Fisher’s exact test or z test

**Table 2:** Tracheal imaging characteristics

	Healthy Individuals (*n* = 53)	Stable COPD (*n* = 40)	AE COPD (*n* = 64)
Tracheal area ratio (%)	74.5 ± 8.6	57.5 ± 19.8	53.8 ± 19.3
ECAC ^a^ *n* (% of group)	0	14 (35%)	25 (39%)
EDAC^+^ * n* (% of group)	0	10 (25%)	15 (23%)
TBM * n* (% of group)	0	Total = 4 (9%) Concentric: 4	Total = 10 (16%) Sabre 4 Concentric 5 Crescentic 1
Anterior-posterior ratio	74.4 ± 8.8	60 ± 18.1	56.3 ± 18
Lateral ratio	84.2 ± 7.3	76.7 ± 11.8	76.6 ± 11.6

^a^Excessive Central Airway Collapse, ^+^Excessive Dynamic Airway Collapse, defined as a > 50% reduction in tracheal luminal area on expiration. Ratios are calculated by inspiratory value divided by expiratory value

Thirty-day mortality was low, with only one death in the AECOPD group. The most common reasons for not returning for follow up in convalescence were patients being unwilling to visit hospital (*n* = 22), lack of transport or had moved out of the area (*n* = 7), and other serious medical illnesses (*n* = 5).

A reduction of 50% tracheal area is conventionally used to diagnose ECAC [1]. No healthy individuals had ECAC by this parameter but 14/40 (35%) patients with stable COPD had ECAC (Table 2). During AECOPD 25/64 (39%) patients had ECAC. The proportion of each group with ECAC did not significantly differ between stable COPD (14/40, 35%) and AECOPD (25/64, 39%, *p* = 0.835). Grouping patients with ECAC into those with EDAC (*n* = 21) or TBM (*n* = 4) did not reveal any differences in anthropometric or clinical characteristics [15].

**Fig. 1:**
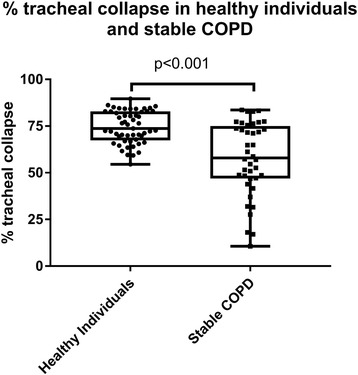
Percentage tracheal collapse in healthy individuals and stable COPD. The extent of tracheal collapse is significantly different in healthy individuals when compared to stable COPD. Tracheal collapse was measured as the ratio of expiratory/inspiratory airway area and expressed as a percentage (%)

Tracheal ratios on expiration (expressed as percentage of minimum expiratory area to maximum inspiratory area) were 74.5 ± 8.6% in healthy individuals, and were significantly lower in stable COPD (57.5 ± 19.8%, 95% CI of difference 10.2 to 23.7%, *p* < 0.001). Tracheal area ratios were also lower in AECOPD (53.8 ± 19.3%, 95% CI of difference 15.2 to 26.0%, *p* < 0.001) (Fig. 1) than in healthy individuals. Tracheal area in AECOPD was similar to stable COPD and did not differ significantly (*p* = 0.355).

We examined if ECAC during AECOPD was associated with adverse outcomes. Patients with AECOPD and ECAC had similar baseline FEV_1_, did not require more frequent non-invasive ventilation and had similar length of stay when compared to individuals without ECAC (Table 3). The clinical severity of exacerbations as judged by severity scores using BAP-65 [15], and admission dyspnoea score (modified Medical Research Council Score [18]) did not differ between these groups (*p* = 0.889 and *p* = 0.152 respectively).

**Table 3:** Comparison of patients with AECOPD with and without ECAC

	No ECAC (*n* = 39)	ECAC (*n* = 25)	*P* value
Age, years	68 ± 12.5	73.4 ± 9.2	0.069
Gender (female:male)	15:24	9:16	1.0
Body mass index (kg/m^2^)	26.7 ± 7.4	25.6 ± 5	0.613
FEV_1_ (% predicted)	48.4 ± 24.7	47.0 ± 21.2	0.843
FVC (% predicted)	75.3 ± 20.4	70.8 ± 20.8	0.488
FEV_1_/FVC (% predicted)	47.0 ± 18.2	48.7 ± 14.1	0.750
Bronchodilator response	8.0 ± 8.5	4 ± 8	0.162
Pack years smoked	60.6 ± 54.7	63.3 ± 58.8	0.880
TLCO (% predicted)	42.2 ± 18.2	48.1 ± 19.7	0.357
Length of stay (days)	4.4 ± 3.3	5.7 ± 6	0.267
Baseline mMRC dyspnea score	2.2 ± 1.2	2.4 ± 1.2	0.404
Admission mMRC dyspnea score	3.7 ± 0.5	3.7 ± 0.4	0.766
BAP65 (class - n)	I 6 II 17 III 12 IV 2	I 1 II 8 III 9 IV 5	0.172
Oral prednisolone at admission (mg/day)	3.9 ± 10.5	1.0 ± 2.2	0.201
Days on noninvasive ventilation	0.3 ± 0.5	2.2 ± 2.6	0.197
Need for noninvasive ventilation (*n*)	4	4	1.0
Death at 30 days (*n*)	1	0	1.0
Hospital admissions in prior 12 months	0.6 ± 1.3	1.0 ± 1.7	0.342
TBM	Sabre 3 Concentric 2 Crescentic 1	Sabre 1 Concentric 3 Crescentic 0	0.732

*ECAC* expiratory central airway collapse; *FEV*_*1*_ forced expiratory volume in one second; *FVC* forced vital capacity; *TLCO* transfer capacity for carbon monoxide; *mMRC* modified Medical Research Council dyspnoea score; BAP65 score – calculated per Shorr et al. [15]. *TBM* tracheobronchomalacia. Data are mean ± SD unless otherwise specified. *P* values are for independent samples t-test or Fisher’s exact test

Using a stricter measure for diagnosis of ECAC (75% tracheal area reduction) reduced patient numbers with ECAC to 10/40 (25%) in the stable COPD group and 7/64 (11%) in the AECOPD group. Again, there were no significant differences in important clinical characteristics and outcomes between groups (Additional file 1: Table S1) [15]. Results were similar if an 80% threshold was adopted for ECAC (not shown). ECAC in both stable COPD and AECOPD groups resulted from posterior membranous collapse as reflected by reductions in antero-posterior proportions (ratio 59.1 ± 18.6%), rather than from reductions in lateral diameter (ratio 78.5 ± 11.8%).

**Fig. 2:**
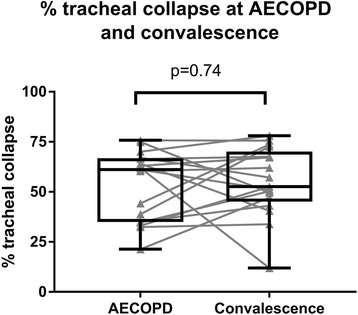
Percentage tracheal collapse in acute exacerbations of COPD and at convalescence. In the subset of patients who returned for repeat CT scans at convalescence (*n* = 17), tracheal collapse is similar between acute exacerbations and convalescence. Tracheal collapse was measured as the ratio of expiratory/inspiratory airway area and expressed as a percentage (%)

Patients with AECOPD were studied by repeat imaging when symptomatically recovered (*n* = 17, clinical characteristics detailed in Additional file 2: Table S2). Mean levels of ECAC in this convalescent group (ratio 54.1 ± 18.9%) were not significantly different to that when they experienced an acute COPD exacerbation (ratio 53.2% ± 17.3%, *p* = 0.742) Fig. 2).

## Discussion

This study demonstrates that significant tracheal instability with partial collapse (ECAC) is substantially more prevalent in patients with stable COPD than in healthy individuals. The prevalence of ECAC was high (35%), was almost identical in AECOPD and remained the same after recovery from AECOPD. These findings indicate that tracheal dysfunction merits consideration as part of comprehensive assessment and management of COPD.

Abnormal changes in tracheal shape and function are well described in COPD [1]. However, studies have been few and the impact on symptoms, airway function, exacerbations and other outcomes are not fully understood. Recent developments in dynamic CT imaging have permitted accurate measurements of tracheal shape and function. Using these techniques we have demonstrated EDAC in COPD [5] as well as abnormal laryngeal movement in severe asthma [19]. The current studies used dynamic, high-speed CT of the trachea to detect and quantify tracheal abnormalities in COPD.

With tidal breathing and dynamic CT, we found ECAC in approximately one third of patients but not in any healthy individuals. Studies that used a forced expiratory technique report a prevalence of ECAC of 53% - 75% when a 50% threshold for diagnosis is applied [2, 20, 21]. However, during forced expiration it is possible that tracheal collapse may occur and overestimate of the presence of ECAC and it has been proposed that ECAC may only be clinically relevant if displayed during tidal breathing [1]. In the COPDGene cohort which used static imaging at functional residual capacity in the majority of patients, and applied a 50% threshold for diagnosis, the prevalence of ECAC in COPD was 5.9% [10].

Our study detected ECAC during tidal respiration rather than during breath-hold or forced expiration and our method represents a dynamic measurement of patho-physiological events. The dynamic nature of our CTs may have given us more time points at which to capture ECAC, resulting in a prevalence that is intermediate between the studies that employed forced expiration, and those that employed paired static imaging. Overall, our study adds to the literature indicating that ECAC is prevalent in COPD and raises the possibility that the abnormality may contribute to additional airway symptoms and possibly other adverse outcomes. For example, smokers in the COPDGene cohort with ECAC had worse respiratory quality of life than those without ECAC [10]. The notion is also supported by a recent study that found that central airway stabilization by way of tracheal stenting or tracheobronchoplasty provided significant symptomatic benefit in COPD across all GOLD stages [22].

Since heightened respiratory effort causes positive intrathoracic pressure during AECOPD, we hypothesized tracheal collapse would be enhanced during AECOPD. However, stable COPD and AECOPD had an almost identical prevalence of ECAC (35% and 39% respectively). This finding was somewhat surprising and may have resulted from inherent constraints associated with imaging studies in AECOPD. CT imaging could only be done once patients’ acute symptoms had stabilised and when they were able to be recumbent. Conceivably more severe ECAC that had been present at the height of symptoms may have improved by the time CT was done and we cannot exclude failing to detect more severe ECAC in this setting. Other measures support our findings. There were no significant differences in clinical characteristics between patients with and without ECAC detectable as gauged by severity of AECOPD (BAP65 scores), dyspnea scores, hospital length of stay, need for non-invasive ventilation and hospitalization in the prior year. Taken together, our findings, the first to evaluate ECAC in AECOPD, suggest that the abnormality is unlikely to have severe and lasting impacts during and following AECOPD - although ECAC may contribute to significant symptoms in stable COPD [1].

A subgroup of patients who were willing to have repeat CT after recovery from AECOPD were also evaluated. This group had similar characteristics to the overall COPD groups and again we found no differences in ECAC between AECOPD and stable COPD. This finding, based on comparing matched CT imaging in the same patients, confirms findings in the stable and AECOPD groups but needs further studies with larger patient numbers to confirm these initial observations.

Our studies have a number of other caveats. The inability to detect differences in ECAC between stable COPD and AECOPD groups and between AECOPD and COPD at convalescence may represent type II error. This appears unlikely since the extent and prevalence of ECAC was virtually identical in the groups. Not all patients with AECOPD were followed up at convalescence, which may reflect selection bias due to a healthy individual effect but again this seems unlikely to have affected outcomes. Boiselle et al. (ref) found no association between ECAC and FEV_1_, pulmonary function measures, quality of life measured by St George’s Respiratory Questionnaire or 6 min walk test (CITE). We did not obtain this data but collected other important acute clinical parameters such as length of non-invasive ventilation, length of hospital stay and BAP-65 were not different between patients with and without ECAC. Since this was a pragmatic observational study we did not measure physiological parameters such as that obtained by pneumotachography, nor were we able to gather chest CT data such as emphysema index or wall area. Finally, although patients with COPD and AECOPD were somewhat older than healthy individuals, it was notable that none of the 12 individuals in the healthy group over the age of 65 had ECAC.

## Conclusions

ECAC may occur in up to one third of patients with stable COPD and the abnormality does not appear to be worsened in AECOPD. Further studies should focus on possible causes of ECAC and whether there is a predisposition to AECOPD in patients known to have ECAC. Clinicians caring for patients with COPD should consider ECAC as a possible contributing factor to breathlessness and other airway symptoms.

## Additional files

Additional file 1: Table S1. Comparison of patients with AECOPD with and without ECAC (ECAC diagnosed by 75% area reduction threshold). (DOCX 17 kb)

Additional file 2: Table S2. Demographics of acute exacerbation of COPD (AECOPD) and COPD at convalescence. (DOCX 16 kb)

## Abbreviations

AECOPD: Acute exacerbation(s) of chronic obstructive pulmonary disease
COPD: Chronic obstructive pulmonary disease
CT: Computed tomography
ECAC: Expiratory central airway collapse
EDAC: Excessive dynamic airway collapse
TBM: Tracheobronchomalacia
