# Supplementary material for: Expiratory central airway collapse in stable COPD and during exacerbations
Source: Respir Res. 2017 Aug 25;18:163. doi: 10.1186/s12931-017-0646-2 (PMC5574204; doi:10.1186/s12931-017-0646-2)
Supplement: Supplementary file 1 — Comparison of patients with AECOPD with and without ECAC (ECAC diagnosed by 75% area reduction threshold). (DOCX 17 kb) [file 12931_2017_646_MOESM1_ESM.docx]

**Additional file 1: Table S1: Comparison of patients with AECOPD with and without ECAC (ECAC diagnosed by 75% area reduction threshold)**

|  | No ECAC  (n=57) | ECAC  (n=7) | P value |
| --- | --- | --- | --- |
| Age, years | 71.6±11 | 58.9±10 | <0.01 |
| Gender (female:male) | 22:35 | 2:5 | 0.70 |
| Body mass index (kg/m^2^) | 26±6.6 | 27.9±4.6 | 0.59 |
| FEV1 (% predicted) | 47.1±22.3 | 54.5±31.6 | 0.54 |
| FVC (% predicted) | 72.2±19 | 83.5±33 | 0.29 |
| FEV_1_/FVC (% predicted) | 47.7±16.8 | 48.5±13 | 0.92 |
| Bronchodilator response | 5.6±8.1 | 12.8±10 | 0.11 |
| Pack years smoked | 62.8±58 | 52.5±33 | 0.73 |
| TLCO (% predicted) | 43.6±19.2 | 52.5±14.4 | 0.37 |
| Length of stay (days) | 5±4.7 | 3.3±2.4 | 0.38 |
| Baseline mMRC dyspnea score | 2.3±1.2 | 2±1.4 | 0.57 |
| Admission mMRC dyspnea score | 3.7±0.4 | 3.5±0.5 | 0.22 |
| BAP65 (class - n) | I 6  II 22  III 19  IV 7 | I 1  II 3  III 2  IV 0 | 1.0 |
| Oral prednisolone at admission (mg/day) | 3.1±8.8 | 0±0 | 0.40 |
| Days on noninvasive ventilation | 1.4±2.2 | 0±0 | 1.0 |
| Need for noninvasive ventilation (n) | 5 | 0 |  |
| Death at 30 days (n) | 1 | 0 | 1.0 |
| Hospital admissions prior 12 months | 0.8±1.6 | 0.1±0.4 | 0.24 |
| TBM | Sabre 3  Concentric 5  Crescentic 1 | Sabre 1  Concentric 0  Crescentic 0 | 0.50 |

ECAC – expiratory central airway collapse. FEV_1_ – forced expiratory volume in one second. FVC – forced vital capacity. TLCO – transfer capacity for carbon monoxide. mMRC – modified Medical Research Council dyspnoea score. BAP65 score – calculated per Shorr et al^17^. TBM – tracheobronchomalacia. Data are mean±SD unless otherwise specified.
