# Supplementary material for: Expiratory central airway collapse in stable COPD and during exacerbations
Source: Respir Res. 2017 Aug 25;18:163. doi: 10.1186/s12931-017-0646-2 (PMC5574204; doi:10.1186/s12931-017-0646-2)
Supplement: Supplementary file 2 — Demographics of acute exacerbation of COPD (AECOPD) and COPD at convalescence. (DOCX 16 kb) [file 12931_2017_646_MOESM2_ESM.docx]

**Additional file 2: Table S2:** **Demographics of acute exacerbation of COPD (AECOPD) and COPD at convalescence**

|  | AECOPD  (n=64) | COPD at convalescence  (n=17) |
| --- | --- | --- |
| Age, years | 70.2±11.6 | 75.5±3.9 |
| Sex, (female:male) | 24:40 | 1:16 |
| Body mass index (kg/m^2^) | 26.2±6.4 | 24.0±6.3 |
| Pack years smoked | 61.8±55.9 | 47±12 |
| FEV_1_ (% predicted) | 47.8±22.9 | 39±12 |
| FEV_1_/FVC | 47.8±16.3 | 40±11 |
| Bronchodilator response (% change) | 6.4±8.5 | 10±9 |
| TLCO (% predicted) | 44.6±18.8 | 38±17 |
| Medications (n) |  |  |
| - Prednisolone 1-10mg/d | 5 | 1 |
| - Prednisolone >10mg d | 3 | 2 |
| - Inhaled CS <= budesonide 800mg/d | 18 | 4 |
| - Inhaled CS > 800mg/d | 39 | 11 |
| - LABA | 56 | 15 |
| - LAMA | 53 | 17 |
| Exacerbations requiring hospital admission in preceding year (n) | 0.77±1.5 | 0.65±1.4 |

FEV_1_ – forced expiratory volume in one second. FVC – forced vital capacity. TLCO – transfer capacity for carbon monoxide. Inhaled CS – inhaled corticosteroid. LABA – long active beta agonist. LAMA – long acting muscarinic antagonist. Data are mean±SD unless otherwise specified. P values are for independent samples t-test, Fisher’s exact test or z test
